# A Novel, Open Access Method to Assess Sleep Duration Using a Wrist-Worn Accelerometer

**DOI:** 10.1371/journal.pone.0142533

**Published:** 2015-11-16

**Authors:** Vincent T. van Hees, Séverine Sabia, Kirstie N. Anderson, Sarah J. Denton, James Oliver, Michael Catt, Jessica G. Abell, Mika Kivimäki, Michael I. Trenell, Archana Singh-Manoux

**Affiliations:** 1 MoveLab – Physical activity and exercise research, Institute of Cellular Medicine, Newcastle University, Newcastle upon Tyne, United Kingdom; 2 Netherlands eScience Center, Amsterdam, The Netherlands; 3 Department of Epidemiology & Public Health, University College London, London, United Kingdom; 4 Regional Sleep Service, Freeman Hospital, Newcastle upon Tyne, United Kingdom; 5 Centre for Research in Epidemiology and Population Health, INSERM, Unit 1018, Villejuif, France; University of Geneva, SWITZERLAND

## Abstract

Wrist-worn accelerometers are increasingly being used for the assessment of physical activity in population studies, but little is known about their value for sleep assessment. We developed a novel method of assessing sleep duration using data from 4,094 Whitehall II Study (United Kingdom, 2012–2013) participants aged 60–83 who wore the accelerometer for 9 consecutive days, filled in a sleep log and reported sleep duration via questionnaire. Our sleep detection algorithm defined (nocturnal) sleep as a period of sustained inactivity, itself detected as the absence of change in arm angle greater than 5 degrees for 5 minutes or more, during a period recorded as sleep by the participant in their sleep log. The resulting estimate of sleep duration had a moderate (but similar to previous findings) agreement with questionnaire based measures for time in bed, defined as the difference between sleep onset and waking time (kappa = 0.32, 95%CI:0.29,0.34) and total sleep duration (kappa = 0.39, 0.36,0.42). This estimate was lower for time in bed for women, depressed participants, those reporting more insomnia symptoms, and on weekend days. No such group differences were found for total sleep duration. Our algorithm was validated against data from a polysomnography study on 28 persons which found a longer time window and lower angle threshold to have better sensitivity to wakefulness, while the reverse was true for sensitivity to sleep. The novelty of our method is the use of a generic algorithm that will allow comparison between studies rather than a “count” based, device specific method.

## Introduction

Large-scale studies have traditionally assessed physical activity and sleep by self-report but objective measurement tools have become increasingly common in the last decade. Studies of physical activity use a hip- or waist-mounted accelerometer that are not suitable for sleep assessment as they are removed prior to bedtime [1]. In sleep studies, parameters are primarily assessed over several days using a 24 hour wrist-mounted accelerometer, commonly known as *actigraphy*[2]. In both areas of research, body acceleration is expressed in manufacturer specific ‘count’ values over a specific time window, called epoch. This approach has limitations as estimates from different accelerometers are not comparable and the analyst has limited control over signal processing [3].

Technological advances in accelerometers over recent years now allow collection of high resolution data in universal units of gravitational acceleration. This type of data, also referred to as raw accelerometry, increases analytical freedom and is more amenable to methodological consistency between studies [3]. The wrist-worn version of such accelerometers has become popular, especially in large population studies [4–7], as compliance is equal or better than waist-worn devices [8–10] and it allows assessment of both physical activity and sleep. A number of algorithms have been developed to derive physical activity variables from raw accelerometer data [6,11,12]. In contrast, relatively little has been done on the extraction of sleep parameters from these data [13,14].

The primary signal used to characterise sleep is lack of body movement, but this judgement is complicated by the fact that there are minor body movements during sleep and [15,16] absence of body movement is also possible during periods of wakefulness. Consequently, sleep characterised by accelerometry primarily represents a sustained lack of body movement when the participant reports being in bed or asleep and may not necessarily result in the same sleep classification as the one from polysomnography or self-reported sleep [15]. Polysomnography, the gold standard for assessment of sleep is a multi-parametric test of biophysiological changes that occur during sleep [17] and requires a laboratory setting, making it expensive and infeasible for large scale studies.

Previous work on sleep detection from accelerometer data relied on algorithms that use magnitude of acceleration as their input [13,18,19]. We propose a novel method, an easier to interpret description of body kinematics, using accelerometer derived arm angle to detect sleep. We also evaluate the agreement of sleep duration parameter derived from this method with self-reported sleep duration in a large sample of community dwelling older adults. Finally, we investigate the impact of method configuration and individual factors such as socioeconomic position, reports of sleep disturbances and depression on agreement between self-reported sleep duration and two accelerometer-assessed sleep parameters, time in bed and total sleep duration. In order to validate our sleep detection algorithm we compared it with data from a polysomnography study in a sample of 28 sleep clinic patients.

## Materials and Methods

### Study Population

Data are drawn from the Whitehall II Study, established in 1985/88, as previously described [20]. Accelerometer measurement was added to the study at the 2012/13 wave of data collection for participants seen at the central London clinic and for those living in the South-Eastern regions of England who underwent a clinical evaluation at home [6]. We used a second study on sleep clinic patients in order to validate our sleep detection algorithm against polysomnography. These data come from 28 adult patients who were scheduled for a one night polysomnography (PSG) assessment at the Freeman Hospital, Newcastle upon Tyne, UK, as part of their routine clinical assessment and were subsequently invited to participate in the study.

### Ethics Statement

In both studies participants were provided with instructions and an information sheet about the study and were given time to ask questions prior to providing written informed consent. The studies were approved by the University College London ethics committee and the NRES Committee North East Sunderland ethics committee, respectively.

### Instrumentation

Participants in the Whitehall II Study had no contraindications (allergies to plastic or metal, travelling abroad the following week) and were asked to wear a tri-axial accelerometer (GENEActiv, Activinsights Ltd, Kimbolton, UK) on their non-dominant wrist for nine (24-h) consecutive days. They were asked to complete a simple sleep log every morning which consisted of two questions: ‘what time did you first fall asleep last night?’ and ‘what time did you wake up today (eyes open, ready to get up)?’ The accelerometer was configured to collect data at 85.70 Hz with a ±8*g* dynamic range. A more elaborate description of the accelerometer protocol can be found in a recent publication [6].

In the second study, polysomnography (Embletta^®^, Denver) was performed using a standard procedure, including video recording, a sleep electroencephalogram (leads C4-A1 and C3-A2), bilateral eye movements, submental EMG, and bilateral anterior tibialis EMG to record leg movements during sleep. Respiratory movements were detected with chest and abdominal bands measuring inductance, airflow was detected with nasal cannulae measuring pressure, and oxygen saturation of arterial blood was measured. Airflow limitation and changes in respiratory movement were used to detect increased upper-airway resistance. All respiratory events and sleep stages were scored according to standard criteria so that EEG determined total sleep time could be measured [15]. Participants were asked to wear the same brand of accelerometer as in the first study (GENEActiv, Activinsights Ltd, Kimbolton, UK) on their non-dominant wrist throughout the one night polysomnography assessment. Here, the accelerometer was also configured to record at 85.70 Hz.

### Data Processing

#### Sleep detection algorithm

**Fig 1:**
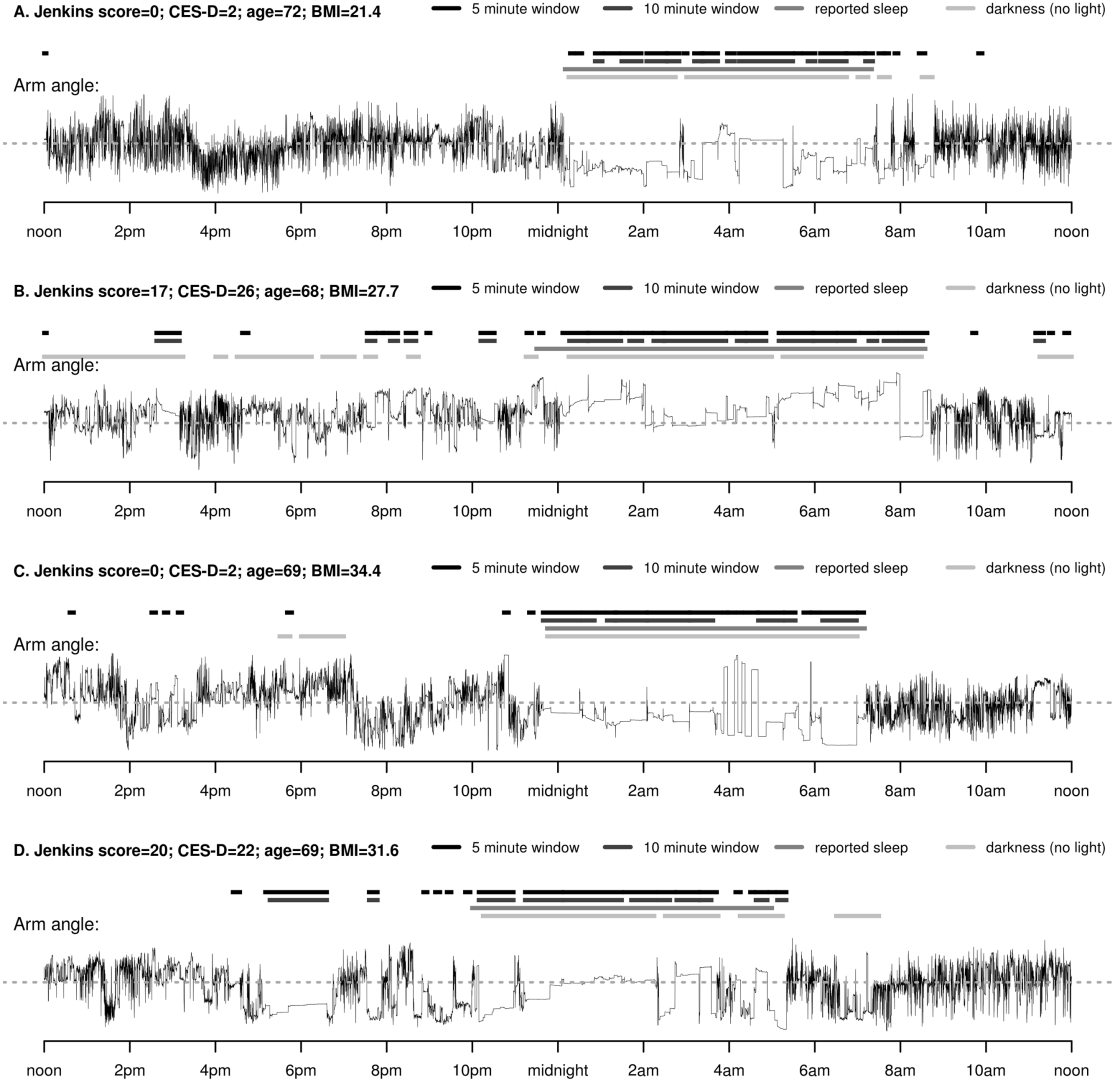
Example of arm angle, detected sustained inactivity bouts, reported sleep and detected darkness from light sensor from the wrist monitor in four participants with distinct individual characteristics (A-D), all on a Thursday evening.

Wrist-worn accelerometer data allow estimation of the arm angle relative to the horizontal plane. By visualizing the arm angle, sleep is characterised as a period marked by a low frequency of changes in arm angle, see thin black line in Fig 1. The accelerometer also had a light sensor (Fig 1) but we decided not to use it for sleep detection as despite its appeal for visual interpretation it can be misleading when the light sensor is covered by a sleeve or bedding.

Previously published methods were used to minimize sensor calibration error [21] and to detect and impute accelerometer non-wear periods [12]. Arm angle was estimated as follows: $angle=\left({\text{tan}}^{-1}\frac{a_{z}}{\sqrt{a_{x}{}^{2}+a_{y}{}^{2}}}\right)\cdot 180/\pi$, where *a*_*x*_, *a*_*y*_ and *a*_*z*_ are the median values of the three orthogonally positioned raw acceleration sensors in g-units (1*g* = 1000 m*g*) derived based on a rolling five second time window. Here, the z-axis corresponds to the axis positioned perpendicular to the skin surface (dorsal-ventral direction when the wrist is in the anatomical position). Next, estimated arm angles were averaged per 5 second epoch, and used to assess change in arm angle between successive 5 second epochs. Periods of time during which there was no change larger than 5° over at least 5 minutes were classified as bouts of sustained inactivity, or potential sleep periods. To demonstrate how a different parameter selection impacts the results, all analyses were replicated with a 10 minute time window criterion. Please see the discussion section for further details on this method.

In the Whitehall II Study summary variables were extracted per 24 hour time window to create a standardised time interval, in this case from noon to noon the following day. However, if the sleep log indicated that the participant was a day sleeper (potential night worker) with sleep onset before noon and a wake up time after noon then a time window from 6pm to 6pm was used. The first and ninth night (24h) were excluded from the analysis, leading to a maximum of seven nights per person in the analysis. Additionally, nights with less than 16 hours of detected wear time were excluded from the analysis. The sleep detection algorithm resulted in one or more sustained inactivity bouts detected per day and for each bout, the duration, the time of sleep onset, and the time of waking were recorded.

Upon publication of this paper our method will be made freely available as an expansion of open-source R-package *GGIR* on CRAN (https://cran.r-project.org)[12,21].

#### Sleep detection aided by sleep log

In the Whitehall II Study a sleep log was used to distinguish nocturnal sleep from bouts of sustained inactivity during the day, which could be related to physical inactivity or day time naps. Sustained inactivity bouts were considered to be nocturnal sleep if they overlapped with a sleep window defined by the sleep log. The following variables were derived: *sleep onset time*, defined as the start of the first nocturnal sustained inactivity bout; *waking time*, defined as the end of the last nocturnal sustained inactivity bout; *time in bed*, defined here as the difference in time between the sleep onset and waking time; and *total sleep duration*, the sum of all nocturnal sleep bouts. Participants who did not have data for at least one weekday and one weekend day were excluded from all analysis that relied on weekly averages of sleep estimates. Average weekly accelerometer assessed sleep parameters were derived as [(average value for weekdays × 5 + average value for weekend days × 2) / 7].

In the polysomnography study the window of sleep classification was not determined by sleep log, but by the more reliable polysomnography recording of total sleep period, which is defined from lights out till waking up.

#### Processing of sleep log

The (visual) screening of accelerometer (actigraphy) data and sleep log data can be cumbersome in large datasets and it complicates replication [22]. Therefore, it is important to automate the method as much as possible in order to allow replication and validation of findings [22,23]. A one dimensional graph showing the overlap between sleep log entries and accelerometer detected sustained inactivity bouts was generated for all nights where the sleep onset time and/or waking time deviated by more than four hours from the nearest accelerometer detected sustained inactivity bout. This allowed us to visualise outliers and assess whether obvious mistakes had been made in the sleep log entry by the participant. These included: confusion between AM and PM, sleep onset and waking time being swapped, or a combination of the two. Visual inspection of 203 nights identified as outliers (out of 28005) resulted in a correction of 33 individual nights across 21 participants’ log entries. Additionally, we found 24 nights across 20 participants with ambiguous log entries, who were subsequently excluded from the analyses. In total 27981 nights from 4094 participants were used in the analysis.

### Covariates

The following measures, drawn from the health survey questionnaire, were included in the analysis:

- *Occupational position at age 50 years* assessed using a 3-level variable (high, intermediate and low) representing income and status at work.
- *Self-reported sleep duration* assessed using the question ‘how many hours of sleep do you have on an average week-night?’. This question was asked a few days ahead of the accelerometer and sleep log assessment to avoid bias resulting from knowledge about sleep log entries.
- *Insomnia symptoms* derived from the Jenkins Sleep Problem Scale [24]. Participants were asked how often in the past month they had experienced the following symptoms: trouble falling asleep, waking up several times per night, trouble staying asleep (including waking far too early), and disturbed or restless sleep. The response scale ranged from 0 (“not at all”) to 5 (“22–31 days”). The sum of individual scores was then categorised as follows: 0, 1–11, 12–20 [25].
- *Depressive symptoms* assessed using the 20-item *Center for Epidemiologic Studies Depression (CES-D) scale*. Scores range between 0 and 60 with higher scores indicating greater depressive symptoms; scores ≥16 were used to represent cases of CES-D depression [26].

In addition, *body mass index* (BMI) was calculated as weight (kilograms) divided by height (meters) squared. Weight was measured by a trained nurse in underwear to the nearest 0.1 kg on Soehnle electronic scales with digital readout (Leifheit AS, Nassau, Germany) and height was measured in bare feet to the nearest 1 mm using a stadiometer with the participant standing erect with head in the Frankfurt plane.

### Statistical analysis

The agreement between weekly average accelerometer assessed sleep duration (time in bed, and total sleep duration − rounded to the nearest integer) and self-reported sleep duration from the health survey questionnaire was assessed using a confusion matrix and a weighted Cohen’s Kappa coefficient [27]. In order to see the influence of individual characteristics, we stratified this analysis by the above mentioned covariates: sex, age (< 70y and ≥70y), occupational position, CES-D depression score, Jenkin’s sleep score, BMI, and the day the accelerometer was worn (weekday versus weekend day).

A *P*-value of < 0.05 was considered significant. The agreement between accelerometer-based estimates of sleep and polysomnography estimates of sleep was quantified by the accuracy, sensitivity and specificity of the binary classification of sleep (any sleep stage) and wakefulness, and by the bias (mean ± standard deviation between patients) in estimated sleep duration.

## Results

Of the 4879 participants to whom the accelerometer was proposed in the Whitehall II Study, 388 did not consent and 210 had contraindications. Of the remaining 4281 participants who wore the accelerometer, 4204 (98.2%) had valid accelerometer data (a readable data file). Among them, sleep log data were missing for 80 participants and 30 additional participants did not meet criteria for accelerometer wear time (at least one night with >16h of wear time). Therefore, the assessment of discrepancies between the accelerometer and the sleep log was undertaken in 4094 participants (83.9% of those invited).

Owing to missing data in the health survey questionnaire on depression (N = 240) and/or sleep (N = 83), additional 285 cases were excluded for the analysis comparing accelerometer assessed and self-reported sleep duration. Furthermore, 40 participants were excluded because they did not have at least one weekday and one weekend day of accelerometer data. The resulting 3769 (75% men) participants were on average 69.3 (standard deviation (SD) = 5.7) years old and had a mean BMI of 26.4 (SD = 4.1) kg/m^2^.

Pearson’s correlation coefficient between sleep parameters derived from a 5 and a 10 minute window was 0.91 for time in bed and 0.98 for total sleep duration (both P < .01). Use of a 10 compared to a 5 minute window led to shorter time in bed (difference = -0.24h; 95% Confidence Intervals (CI):-0.29, -0.20) and total sleep duration (difference = -0.94h; 95%CI:-0.99, -0.90).

The median delay between responding to the health survey questionnaire and the first day of accelerometer wear was 5 days (interquartile range 1–22 days). Agreement between questionnaire based sleep duration and accelerometer estimated time in bed was similar for both time windows (Kappa = 0.32 for 5 min and 0.36 for 10 min window). However, agreement between questionnaire based sleep duration and accelerometer estimated total sleep duration was higher for the 5 minute (Kappa = 0.39) than the 10 minute window (Kappa = 0.18) (Table 1). Accelerometer assessed total sleep duration tended to be greater than self-reported sleep duration for short sleepers and smaller for long sleepers; total accelerometer assessed sleep duration was smaller in long sleepers (Table 1).

**Table 1:** Agreement between sleep duration categories estimated by sleep survey questionnaire and by accelerometer methods.

			Sleep survey					Kappa	
			N (%^†^)					Coefficient	95%CI
Accelerometer estimates*			≤5 hrs	6 hrs	7 hrs	8 hrs	≥9hrs		
Time in bed		N	292	1142	1431	799	105		
5 minutes window									
	≤ 5 hrs	53	24 (8%)	27 (2%)	1 (0%)	1 (0%)	0 (0%)	0.32	0.29, 0.34
	6 hrs	315	55 (19%)	193 (17%)	60 (4%)	6 (1%)	1 (1%)		
	7 hrs	1264	109 (37%)	514 (45%)	533 (37%)	104 (13%)	4 (4%)		
	8 hrs	1620	87 (30%)	330 (29%)	683 (48%)	487 (61%)	33 (31%)		
	≥9hrs	517	17 (6%)	78 (7%)	154 (11%)	201 (25%)	67 (64%)		
Mean (SD) in hours	7.6 (0.89)		7.02 (1.08)	7.21 (0.88)	7.66 (0.69)	8.11 (0.66)	8.81 (0.83)		
10 minutes window									
	≤ 5 hrs	104	36 (12%)	57 (5%)	8 (1%)	3 (0%)	0 (0%)	0.36	0.34, 0.39
	6 hrs	458	74 (25%)	245 (21%)	124 (9%)	14 (2%)	1 (1%)		
	7 hrs	1506	106 (36%)	548 (48%)	643 (45%)	199 (25%)	10 (10%)		
	8 hrs	1369	64 (22%)	243 (21%)	570 (40%)	454 (57%)	38 (36%)		
	≥9hrs	332	12 (4%)	49 (4%)	86 (6%)	129 (16%)	56 (53%)		
Mean (SD) in hours	7.36 (0.91)		6.75 (1.09)	6.96 (0.9)	7.42 (0.71)	7.88 (0.68)	8.54 (0.82)		
**Total sleep duration**									
5 minutes window									
	≤ 5 hrs	432	89 (30%)	217 (19%)	97 (7%)	28 (4%)	1 (1%)	0.39	0.36, 0.42
	6 hrs	1212	112 (38%)	484 (42%)	470 (33%)	136 (17%)	10 (10%)		
	7 hrs	1563	75 (26%)	371 (32%)	684 (48%)	394 (49%)	39 (37%)		
	8 hrs	522	15 (5%)	67 (6%)	172 (12%)	225 (28%)	43 (41%)		
	≥9hrs	40	1 (0%)	3 (0%)	8 (1%)	16 (2%)	12 (11%)		
Mean (SD) in hours	6.58 (0.94)		5.97 (1.07)	6.21 (0.92)	6.66 (0.78)	7.07 (0.80)	7.56 (0.88)		
10 minutes window									
	≤ 5 hrs	1586	183 (63%)	641 (56%)	547 (38%)	196 (25%)	19 (18%)	0.18	0.16, 0.20
	6 hrs	1435	81 (28%)	382 (33%)	596 (42%)	339 (42%)	37 (35%)		
	7 hrs	662	27 (9%)	107 (9%)	269 (19%)	231 (29%)	28 (27%)		
	8 hrs	78	1 (0%)	12 (1%)	17 (1%)	30 (4%)	18 (17%)		
	≥9hrs	8	0 (0%)	0 (0%)	2 (0%)	3 (0%)	3 (3%)		
Mean (SD) in hours	5.64 (1.02)		5.13 (1.08)	5.32 (1.01)	5.71 (0.92)	6.05 (0.97)	6.43 (1.06)		

*Accelerometer estimates are rounded to the unity.

^†^Percentages are calculated per column.

The agreement between questionnaire assessed sleep duration and time in bed was lower in women, in depressed participants, in those reporting more insomnia symptoms, and on weekend days (Table 2). However, no such differences were observed for total sleep duration (Table 2). S1–S3 Figs provide a visual representation of the confusion matrices underlying the Kappa coefficients for sex, sleep quality, and depression.

**Table 2:** Agreement between sleep duration categories estimated by sleep survey questionnaire and by accelerometer methods in different subgroups.

		Time in bed				Total sleep duration			
Subgroups		Kappa coefficient				Kappa coefficient			
Window	N	5 minutes		10 minutes		5 minutes		10 minutes	
		Estimate	95%CI	Estimate	95%CI	Estimate	95%CI	Estimate	95%CI
Sex									
Men	2826	0.34	0.31, 0.37	0.39	0.36, 0.42	0.41	0.38, 0.44	0.18	0.16, 0.21
Women	943	0.27	0.22, 0.31	0.30	0.25, 0.35	0.35	0.30, 0.41	0.20	0.15, 0.24
Age									
<70y	2132	0.31	0.28, 0.34	0.37	0.33, 0.40	0.38	0.34, 0.42	0.16	0.14, 0.19
≥70y	1637	0.32	0.29, 0.36	0.36	0.32, 0.40	0.40	0.36, 0.44	0.21	0.18, 0.24
Occupational position at age 50y									
Low	368	0.23	0.17, 0.30	0.29	0.21, 0.37	0.35	0.26, 0.44	0.17	0.09, 0.24
Intermediate	1677	0.33	0.30, 0.37	0.38	0.34, 0.41	0.39	0.35, 0.43	0.19	0.16, 0.22
High	1724	0.32	0.29, 0.36	0.37	0.33, 0.41	0.39	0.35, 0.43	0.18	0.15, 0.21
CES-D score									
<16	3355	0.34	0.32, 0.37	0.38	0.35, 0.41	0.40	0.37, 0.43	0.18	0.16, 0.20
≥16	414	0.20	0.14, 0.26	0.26	0.19, 0.33	0.32	0.23, 0.40	0.22	0.14, 0.30
Jenkins sleep scale									
0	313	0.59	0.51, 0.67	0.59	0.51, 0.66	0.44	0.36, 0.52	0.18	0.13, 0.23
1–11	2855	0.36	0.33, 0.38	0.40	0.37, 0.43	0.40	0.37, 0.43	0.17	0.15, 0.19
12–20	601	0.14	0.11, 0.18	0.19	0.14, 0.23	0.27	0.20, 0.34	0.22	0.14, 0.30
BMI									
< 25 kg / m^2^	1356	0.34	0.30, 0.38	0.38	0.33, 0.42	0.40	0.36, 0.45	0.18	0.14, 0.21
25–29.9 kg / m^2^	1531	0.31	0.27, 0.35	0.36	0.32, 0.41	0.38	0.34, 0.43	0.18	0.15, 0.21
> 30 kg / m^2^	571	0.31	0.25, 0.37	0.35	0.29, 0.42	0.39	0.31, 0.46	0.18	0.13, 0.22
Day									
Weekday (Sun–Thu)	3769	0.33	0.31, 0.35	0.37	0.34, 0.39	0.37	0.34, 0.40	0.17	0.15, 0.19
Weekend day (Fri, Sat)	3769	0.25	0.23, 0.28	0.29	0.26, 0.31	0.33	0.31, 0.36	0.17	0.15, 0.19

All 28 patients recruited for the polysomnography study (11 female) had complete data and were aged between 21 and 72 years (mean±sd: 45±15 years). Of them, 19 had a sleep disorder according to the International Classification of Sleep Disorders. The agreement between accelerometer estimates of sleep using our algorithm and polysomnography derived parameters was good, as shown in Table 3. For example, sleep parameters derived with a 5 minute window and a 5 degree angle threshold had on average a 31 minute overestimation of sleep duration and an 83% accuracy (Table 3).

**Table 3:** Classification agreement between accelerometer and polysomnography.

Time window (minutes)	Angle (degrees)	Accuracy (%)	Sensitivity (%)	Specificity (%)	Bias (minutes)
3	3	83 ± 8	92 ± 13	40 ± 22	40 ± 41
5	3	83 ± 8	88 ± 16	51 ± 24	13 ± 43
7	3	81 ± 11	82 ± 22	58 ± 24	-14 ± 57
10	3	78 ± 13	75 ± 23	65 ± 22	-43 ± 65
15	3	73 ± 15	67 ± 25	72 ± 21	-86 ± 76
3	5	82 ± 9	94 ± 11	34 ± 20	55 ± 38
5	5	83 ± 8	91 ± 13	45 ± 23	31 ± 38
7	5	83 ± 9	86 ± 20	53 ± 24	7 ± 45
10	5	81 ± 10	81 ± 21	60 ± 23	-17 ± 47
15	5	77 ± 12	73 ± 22	65 ± 23	-58 ± 63
3	10	81 ± 8	96 ± 8	28 ± 17	74 ± 44
5	10	83 ± 8	95 ± 9	37 ± 22	53 ± 34
7	10	84 ± 8	92 ± 11	44 ± 23	35 ± 35
10	10	83 ± 8	88 ± 13	51 ± 23	14 ± 40
15	10	80 ± 11	79 ± 20	58 ± 24	-28 ± 61

## Discussion

This paper presents a novel method to detect sleep duration from raw accelerometer data collected using a wrist-worn device and assesses agreement of these parameters with both self-reported sleep duration and polysomnography. We used an accelerometer with 5 minute and 10 minute criteria to detect bouts of sustained inactivity based on change in arm angle; following the standard protocol in sleep studies we distinguished nocturnal sleep from daytime inactivity using a sleep log [28]. Overall there was moderate agreement between accelerometer and questionnaire assessed sleep duration, except for total sleep duration derived with a 10 minute time window configuration for which the agreement was poor. In subgroup analyses, we found lower agreement for time in bed in women, depressed participants, and those reporting more insomnia symptoms. However, no differences in agreement as a function of these variables were observed for total sleep duration.

Previously published methods for sleep detection using accelerometers optimized their parameters to best fit with polysomnography-derived periods of sleep [14,18]. We did not use parameter optimisation, but instead aimed for a method that provided an easy to understand (heuristic) description of wrist kinematics. Two requirements were identified: 1) data should be expressed in universal kinematic units and not in “count” values that are device specific, and not easily interpretable, and 2) method parameters should be derived logically and not result from statistical regressions on a particular sample. Consequently, arm angle was considered easier to interpret than magnitude of acceleration. The selection of a 5° angle threshold based on the median value of a moving 5 second window was assumed to capture all static postures and to filter out minor breathing movements and movements of the bed partner. The 5 and 10 minute window of substantial inactivity criterion were assumed to be plausible time intervals after which sleep becomes more likely. Additional parameter configurations were evaluated in the polysomnography study confirming that a 5 and a 10 minute window together with a 5 degree angle threshold provide informative estimates of sleep.

Previous findings from the comparison between accelerometer estimates of sleep and a concurrently applied sleep log [15,29,30] should be interpreted with caution because data from the log was used to time nocturnal sleep initiation and waking time, boosting the agreement between the two methods. Only a few studies have compared accelerometer derived estimates of sleep with independently collected self-reported sleep duration [31,32]. One study based on 56 adult women (age range 18-80y) found kappa values ranging from -0.19 to 0.14 for different sleep estimates [32]. Another study based on 225 adolescents (age range: 11-13yr) found no significant correlation (r: 0.06; p > .05) between actigraphy and self-reported sleep duration [31]. In the present study, greater agreements were found between accelerometer and questionnaire estimated sleep duration. Better agreement in our data could be explained by the accuracy of our accelerometer method or the age group examined in our study.

For the accelerometer method the time window used to decide that an inactivity bout is sleep (5 vs. 10 minutes) affects the estimated sleep duration, both for time in bed and total sleep duration, with durations being longer with the 5 minute window. The estimated total sleep duration we found for a 5- compared to a 10-minute window was closer to reports from studies using polysomnography in older adults from the general population (range being 6 to 6.7h)[33–36]. In addition, the agreement between self-reported sleep duration and accelerometer-assessed total sleep duration was better for a 5 minute window, while agreement for time in bed was moderate for both 5 and 10 minute time windows. A 10 minute window could be hypothesised to be better adapted for ignoring inactive periods while awake while a 5 minute window may be more sensitive in capturing all sleep periods. The interpretation of the sleep duration question may also affect the level of agreement between the methods. Some participants may have answered the question with their recall of total hours of sleep, while other participants may have interpreted the question about sleep duration as the time difference in hours between sleep onset and waking time, time in bed. Future studies using direct comparison against polysomnography in a larger sample of the general population settings are needed to help inform a decision about time window configuration. Our polysomnography study showed that sensitivity to detect sleep is better for shorter time windows and higher angle thresholds while the specificity to detect sleep, and therefore the sensitivity to detect wakefulness, is better for longer time windows and lower angle thresholds. These results are in line with results of comparison with self-reported sleep as discussed above. The optimal method configuration to minimize bias in estimated sleep duration depends on the true duration of sleep, which is unknown [37]. Therefore, a generic configuration for the optimal setting is likely to reflect the data structure of the derivation sample, which may differ from the data structure of the application sample. Our suggestion is to use knowledge (believes) of the effect of sleep on body posture, in terms of parameters such as arm angle and time of sustained inactivity, rather than count data produced by conventional methods for which optimization in particular datasets is the only pathway for interpretation.

The lower agreement for time in bed seen in women, depressed participants, those reporting more insomnia symptoms, and on weekend days may be explained by a lower validity of self-reported sleep in these groups or on these days of the week, a lower validity of the accelerometer method, or a combination of the two [38]. For example, disturbed sleep may present challenges for both methods: difficulties in estimating sleep duration by oneself, more inactive wake periods that can be confused with sleep, and possibly more minor movements during sleep that can be confused with wakefulness. However, the absence of a noticeable difference in method agreement by individual factors for total sleep duration seems to indicate that the agreement discrepancies are specific to the time in bed parameter.

The main strength of accelerometry is that it can provide complementary estimates of sleep that cannot be derived from self-report methods, e.g. total sleep duration, sleep efficiency, and sleep fragmentation. A specific strength of raw data accelerometry when used on the wrist is that it can be combined with assessment of physical activity using the same device in order to facilitate research on the relationship between sleep and physical activity. Our study findings need to be interpreted in light of some limitations. The sleep log we used was relatively simple. A more detailed sleep log including specific questions for naps, times into and out of bed, and time lights off, may well allow better assessment of sleep. However, the addition of questions may also increase the burden on the participants and result in missing data or incorrect data entries. Future research is needed to compare the accelerometer method with polysomnography in a larger sample to better understand discrepancies between neurological sleep and sleep defined by wrist kinematics.

In conclusion, agreement between sleep duration estimates by our newly developed method for accelerometer data and sleep questionnaire was moderate. We found that the agreement with self-reported sleep duration is least affected by individual factors when sleep duration is defined as total sleep duration over a night.

## Supporting Information

S1 Fig Agreement between questionnaire assessed sleep duration and accelerometer assessed total sleep duration based on a 5 minute (a and b) and a 10 minute (c and d) window, in men (a and c) and women (b and d). (TIFF)

S2 Fig Agreement between questionnaire assessed sleep duration and accelerometer assessed total sleep duration based on a 5 minute (a and b) and a 10 minute (c and d) window, in participants with CES-D score < 16 (a and c) and those with CES-D score ≥ 16 (b and d). (TIFF)

S3 Fig Agreement between questionnaire assessed sleep duration and accelerometer assessed total sleep duration based on a 5 minute (a, b and c) and a 10 minute (d, e and f) window, in participants with Jenkins score = 0 (a and d), Jenkins score between 1–11 (b and e), and Jenkins score between 12–20 (c and f). (TIFF)
