# Supplementary figures and images for: A Novel, Open Access Method to Assess Sleep Duration Using a Wrist-Worn Accelerometer
Source: PLoS One. 2015 Nov 16;10(11):e0142533. doi: 10.1371/journal.pone.0142533 (PMC4646630; doi:10.1371/journal.pone.0142533)

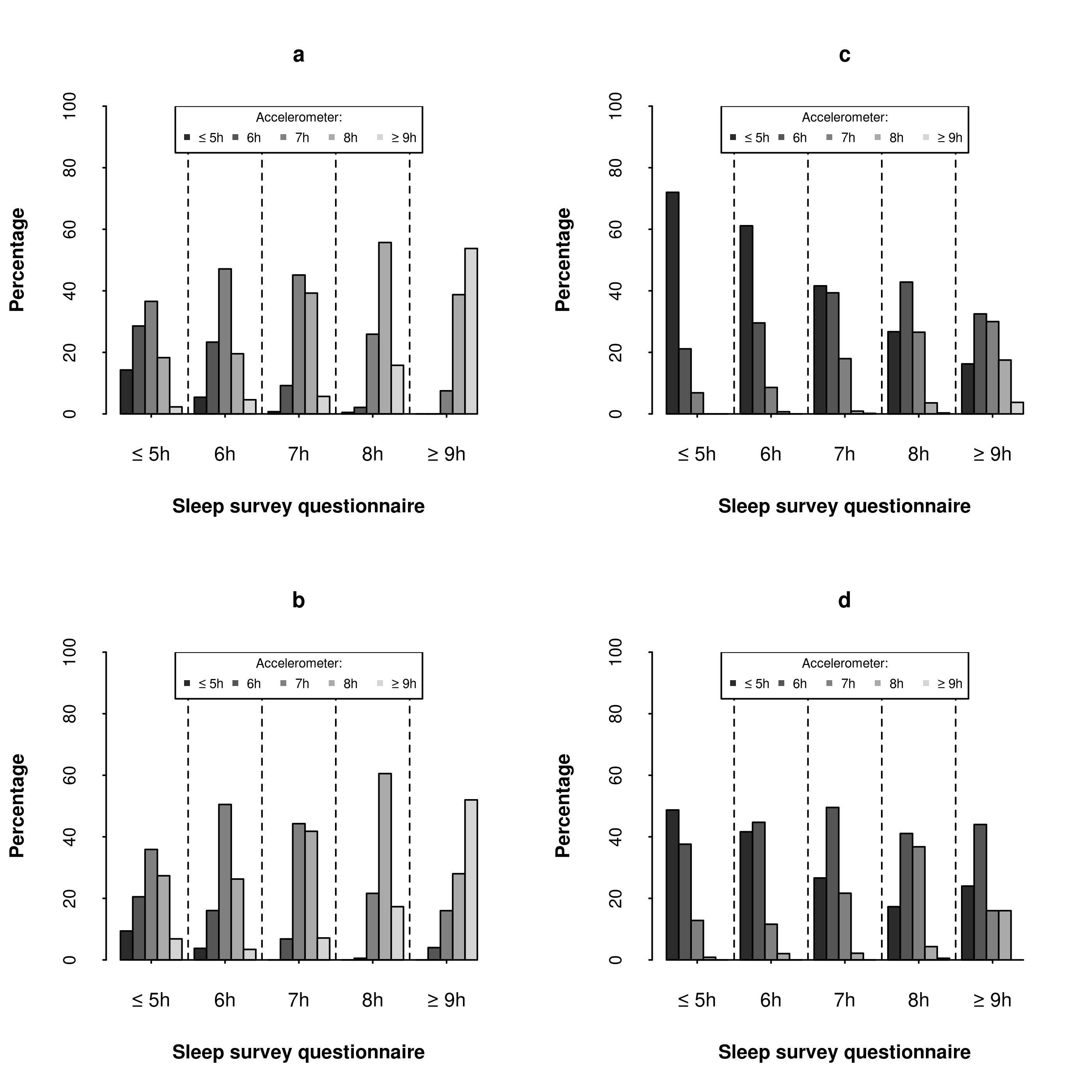

Supplement: S1 Fig — (TIFF) [file pone.0142533.s001.tiff]

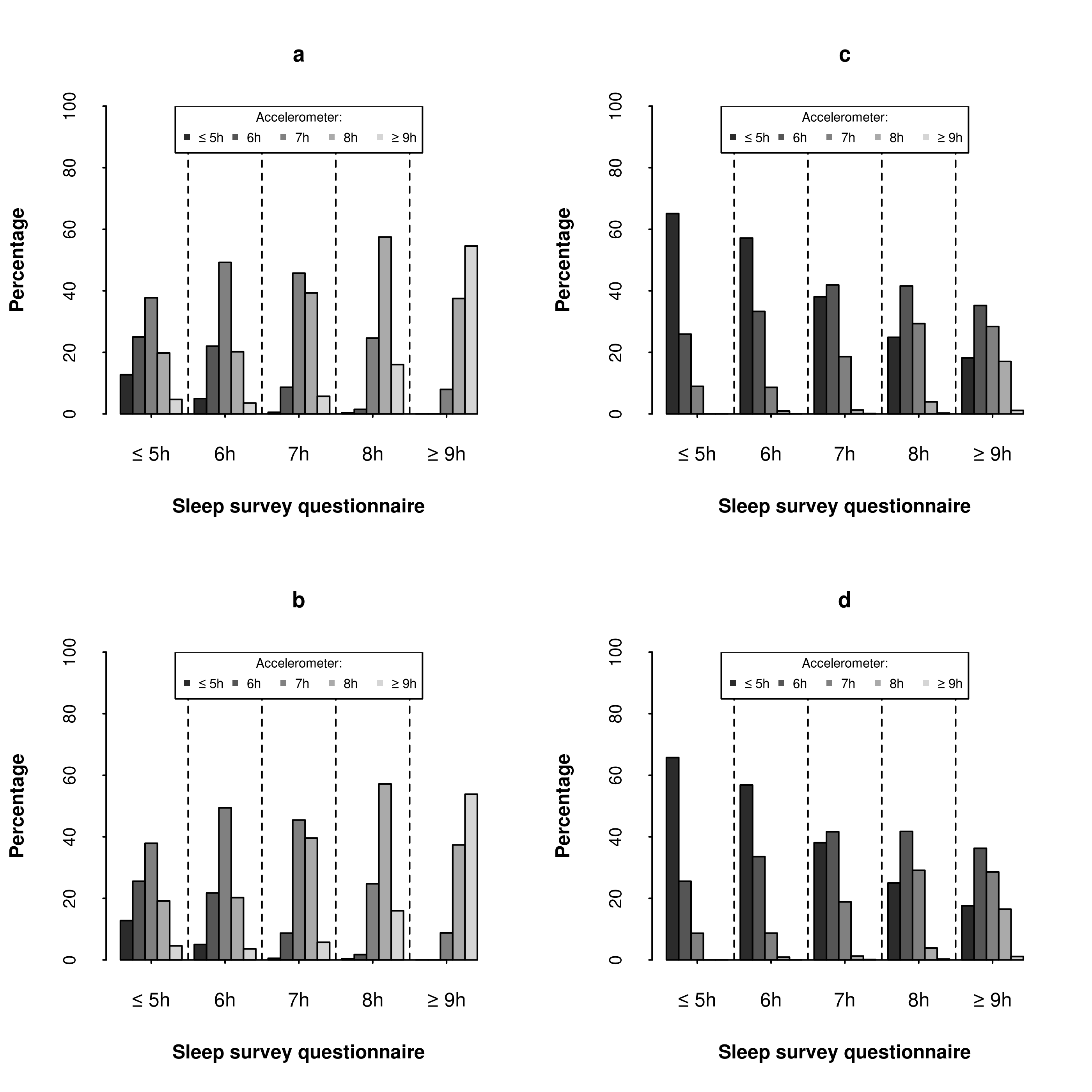

Supplement: S2 Fig — (TIFF) [file pone.0142533.s002.tiff]

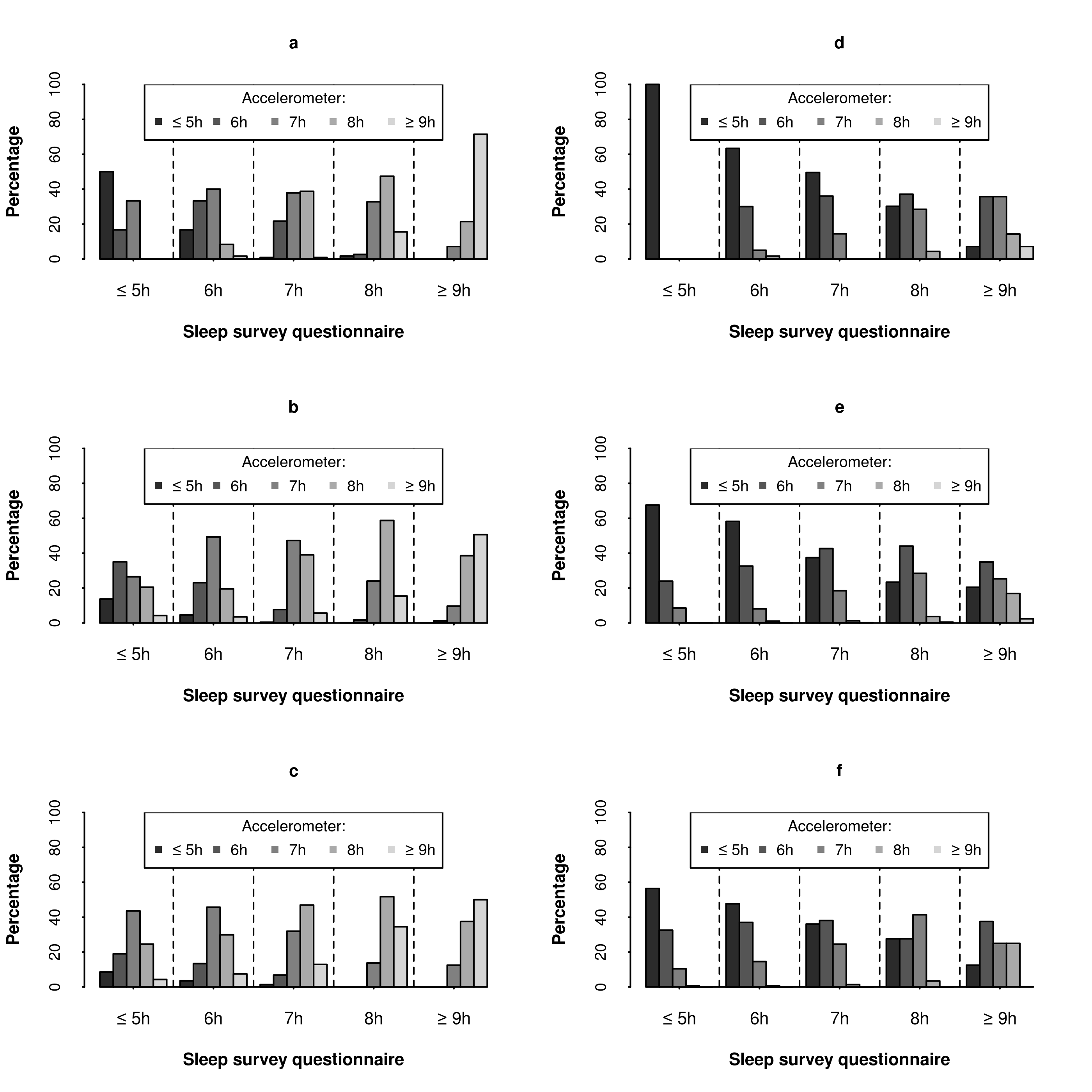

Supplement: S3 Fig — (TIFF) [file pone.0142533.s003.tiff]
